# Micro-Ribonucleic Acid-216a Regulates Bovine Primary Muscle Cells Proliferation and Differentiation *via* Targeting *SMAD Nuclear Interacting Protein-1* and *Smad7*

**DOI:** 10.3389/fgene.2019.01112

**Published:** 2019-11-13

**Authors:** Zhaoxin Yang, Chengchuang Song, Rui Jiang, Yongzhen Huang, Xianyong Lan, Chuzhao Lei, Hong Chen

**Affiliations:** Shaanxi Key Laboratory of Animal Genetics, Breeding and Reproduction, College of Animal Science and Technology, Northwest A&F University, Yangling, China

**Keywords:** miR-216a, *SMAD nuclear interacting protein-1*, *smad7*, skeletal muscle, bovine

## Abstract

MicroRNAs (miRNAs), belonging to a class of evolutionarily conserved small noncoding RNA of ∼22 nucleotides, are widely involved in skeletal muscle growth and development by regulating gene expression at the post-transcriptional level. While the expression feature and underlying function of miR-216a in mammal skeletal muscle development, especially in cattle, remains to be further elucidated. The aim of this study was to investigate the function and mechanism of miR-216a during bovine primary muscle cells proliferation and differentiation. Herein, we found that the expression level of miR-216a both presented a downward trend during the proliferation and differentiation phases, which suggested that it might have a potential role in the development of bovine skeletal muscle. Functionally, during the cells proliferation phase, overexpression of miR-216a inhibited the expression of proliferation-related genes, reduced the cell proliferation status, and resulted in cells G1 phase arrest. In cells differentiation stages, overexpression of miR-216a suppressed myogenic maker genes mRNA, protein, and myotube formation. Mechanistically, we found that *SNIP1* and *smad7* were the directly targets of miR-216a in regulating bovine primary muscle cells proliferation and differentiation, respectively. Altogether, these findings suggested that miR-216a functions as a suppressive miRNA in development of bovine primary muscle cells *via* targeting *SNIP1* and *smad7*.

## Introduction

Skeletal muscle, as an important sports and metabolic organization of animals, accounts for a large proportion of body weight and is directly related to the economic traits such as meat production in cattle. The skeletal muscle development in mammal undergoes two important stages the increase in the number of pre-natal muscle fibers (Russell and Oteruelo, 1981; Hocquette, 2010) and the increase in the volume of muscle fibers after birth (Zhu et al., 2006). Therefore, it is of great value to study the intrinsic mechanism of muscle formation. Skeletal muscle development originates from the embryonic stage, which is derived from the paraxial mesoderm structures in the vertebrate (Christ and Ordahl, 1995). Here, the dorsal somite forms the dermomyotome which can further form the myotome (Tajbakhsh and Buckingham, 2000). Then, the muscle progenitor cells will form and migrate from the myotome. Myotome quickly differentiates into primary myoblasts. Finally, these primary muscle progenitor cells undergo proliferate and differentiate to form skeletal muscle (Buckingham et al., 2003). Orchestral molecules mechanisms are involved in the process of myogenesis, which are mainly regulated by transcription factors likely paired box transcription factors *Pax3/Pax7* (Relaix et al., 2006; Lagha et al., 2008; Buckingham and Relaix, 2015), *myogenic regulatory factors (MRFs)* (Ott et al., 1991), and *myocyte enhancer factor 2 family (MEF2)* (Edmondson et al., 1994). It’s worth noting that these key transfection factors are also regulated by other multiple regulatory factors, including protein coding gene such as *transforming growth factor-beta (TGF-β)* (Liu et al., 2001; Gardner et al., 2011) and noncoding RNA likely microRNAs (miRNAs) (Chen et al., 2006).

MiRNAs are a variety of evolutionarily conserved short noncoding RNA. The function and molecular mechanism of miRNAs has been extensively studied in almost all biological processes. The mature sequence of miRNA could complete or partial complementary pair with the 3’ UTR of mRNA, which cause mRNA decay or impede protein translation (Bartel, 2004; Bartel, 2009). MiRNAs, as an integral part of skeletal muscle development, can regulate skeletal muscle proliferation, differentiation, and regeneration by targeting the key factors of the myogenic regulatory network during the different stages of skeletal muscle development process (Chen et al., 2006; Crist et al., 2009; Cheung et al., 2012; Alexander et al., 2013; Diniz and Wang, 2016). Studies have shown that dicer knock-out mice in embryonic skeletal muscle resulted in embryonic lethality, skeletal muscle hypoplasia, myofiber morphogenesis defects, and increased apoptosis (O'Rourke et al., 2007). So far, large numbers of miRNAs have been report to affect skeletal muscle development (Ge and Chen, 2011). Some of them are muscle specific expression miRNAs (myomiRs), including miR-1 (Chen et al., 2006; Sluijter et al., 2010), miR-133 (Chen et al., 2006; Feng et al., 2013), miR-206 (McCarthy, 2008; Dey et al., 2011), etc. miR-1 could promote myogenic cells differentiation by targeting *HDAC4* (Chen et al., 2006). miR-133 could promote myoblast proliferation by repressing the expression of *SRF* (Chen et al., 2006). In the differentiation stage of satellite cells, the expression of miR-206 is upregulated, which can enhance satellite cell differentiation by targeting *Pax7* (Dey et al., 2011). Besides that, numerous non-muscle-specific miRNAs (non-myomiRs) also play important role in the regulation of myogenesis (Ju et al., 2015). For example, miR-26a can promote myogenesis by targeting transcription factors *Smad1, Smad4,* and *Ezh2 (enhancer of zeste homologue 2)*, a negative regulator of myogenesis (Wong and Tellam, 2008; Dey et al., 2012). miR-486 can promote muscle differentiation by activating *PI3K/AKT* pathway or targets *Pax7* directly (Small et al., 2010; Dey et al., 2011). MiR-22 can inhibit proliferation and enhances differentiation of C2C12 myoblasts by targeting *TGF-βR1* (Wang et al., 2018b). Although the research of miRNAs is receiving considerable, there are still many miRNAs to be further investigated in skeletal muscle.

miR-216a is a widely expressed miRNA that involved in disease and tumorigenesis by targeting different target genes (Ji et al., 2017; Tao et al., 2017; Wang et al., 2017a). However, little is known about the function of miR-216a in regulation of bovine skeletal muscle development. Therefore, the purpose of current study was to explore the function and mechanism of miR-216a for bovine primary muscle cells proliferation and differentiation. Here, the expression level of miR-216a presented a downward trend both in the proliferation and differentiation phases of bovine skeletal muscle cell. Functional analysis found that miR-216a could inhibit the proliferation and differentiation of bovine skeletal muscle cell by directly targeting *SNIP1* and *smad7* respectively.

## Materials and Methods

### Animals and Tissue Sample Collection

Three months old fetal Qinchuan cattle from Shaanxi Kingbull Livestock Co., Ltd. (Baoji, China) were used in this research. The heart, liver, spleen, lung, kidney, stomach, gut, and leg muscle were collected and kept at -80°C until RNA isolation. Animal care and study protocols were approved by the Animal Care Commission of the College of Veterinary Medicine, Northwest A&F University (permit number: NWAFAC1019).

### Cell Culture

Bovine skeletal muscle cells are derived from the longissimus muscle or hind limbs of the 3-month-old fetus. The stripped muscle tissue is cut and then digested with collagenase I. The specific separation steps refer to the previous description (Miyake et al., 2012). The growth stage bovine skeletal muscle cells were cultured in DMEM with 20% FBS and 1% penicillin/streptomycin. When inducing skeletal muscle cells differentiation, replace 20% FBS in the medium with 2% horse serum. HEK293T cells (ATCC, USA) were cultured in DMEM with 10% FBS and 1% penicillin/streptomycin. All these cells were cultured at 37°C in a 5% CO2 atmosphere.

### Plasmid Construction and Cell Transfection

The wild-type and mutant-type sequences of *smad7* or *SNIP1* 3’UTR were inserted into the psi-check2 reporter vector, respectively. The mutant type 3’UTR sequences of *smad7* or *SNIP1* each contained four nucleotide mutations at the miR-216a targeting site. The precursor sequences of miR-216a were cloned into pcDNA3.1 (+) expression vector. The mimic and inhibitor of miR-216a were purchased from Ribobio (Guangzhou, China). The primers used in this study were listed in **Supplementary Table 1**.

The proliferated bovine primary muscle cells were transfected with miR-216a mimic, inhibitor, and control when the cell density reached 60–70% and collected at 24 h after transfection in 12-well plates. The differentiated bovine primary muscle cells were transfected when the cell density reached 70–80% confluence and 20% FBS medium was changed to 2% horse serum to induce cells differentiation at 24 h after transfection. Cells were collected at three days after induced differentiation. The bovine primary muscle cells used in CCk-8, 5-ethynyl-2′-deoxyuridine (EdU) experiment, and HEK293T cells were cultured in 96-well plates, and they were collected at 24 h after transfection. Thermo transfection reagent was used in this study. The detail procedure of transfection was performed according to the manufacturer’s instructions.

### Real-Time Quantitative Polymerase Chain Reaction

Total RNA of tissues and cells was extracted with TRIzol Reagent (Takara) according to the manufacturer’s instruction. Total RNA was reverse transcribed by using the PrimeScript™ RT Reagent Kit with gDNA Eraser (Takara). Real-time quantitative PCR (RT-qPCR) was performed with the SYBR Green Kit (Genestar, Beijing, China) on a CFX96 system (Bio-Rad, Hercules, CA, USA) with three biological replicates each time. Relative expression levels of mRNAs and miRNAs were calculated with the 2^-∆∆Ct^ method (Livak and Schmittgen, 2001). *U6* and *GAPDH* gene was used as reference gene for the expression of miRNA and all genes. The primers used in this study were listed in **Supplementary Table 1**.

### Western Blot

Total proteins were extracted from cells by using radio immunoprecipitation assay buffer with 1% PMSF (Solarbio, Beijing, China). The protein concentration was measured by using the BCA Protein Assay Kit (Beyotime, Shanghai, China) and denatured with 5× protein loading buffer at 98°C for 10 min. The specific operation process of WB was described previously (Li et al., 2018). In short, the target proteins were separated by sodium dodecyl sulfate polyacrylamide gel electrophoresis and transferred to a methanol-activated polyvinylidene fluoride (PVDF) membrane. The corresponding primary antibody was added to incubate PVDF membrane for overnight at 4°C. At room temperature, the PVDF membrane was re-incubated with the corresponding secondary antibody for 2 h. The protein signal strength was detected using enhanced chemiluminescence reagent. The primary antibodies and secondary antibodies were listed in **Supplementary Table 2**. The quantified of the protein was performed by the ImageJ program (National Institutes of Health).

### Cell Proliferation Assay

For the CCK-8 assay, the bovine primary muscle cells were transfected with the negative control (NC), mimics, and inhibitor when the cell densities were 50–60%. After transfection for 24 h, the culture medium containing 10% CCK-8 reagent was changed and incubated the cells for 2 h. The CCK-8 Reagent Kit (Tiandz, Beijing, China) was performed to measure cell proliferation index at 450 nm by using Microplate Reader (Tecan, Switzerland) and repeated five times for each independent experiment. The Cell-Light EdU DNA Cell Proliferation Kit (Ribobio, Guangzhou, China) was used to measure S phase positive cell according to the manufacturer’s instructions. The EdU positive cells was observed by using fluorescence microscope (AMG EVOS, USA) and each treatment group had three independent replicates.

### Cell Cycle Assay

The muscle cells were seeded into 60 mm diameter petri dish and transfected with the NC, mimics, and inhibitor when the cell densities were 60%. After transfection for 24 h, muscle cells were fixed in PBS containing 70% ethanol, and cell cycle assays were performed with cell cycle staining kit (MultiSciences Biotech Co., Ltd, Hangzhou, China) by using a flow cytometry. The experimental procedure is based on our previous research (Song et al., 2015).

### Dual-Luciferase Activity Assay

The target genes of miR-216a were predicted with the online software TargetScan (http://www.targetscan.org). The 3’ untranslated region (3’UTR) (wild-type or mutant-type) sequences of the genes SNIP1 and smad7 mRNA was cloned into the psi-Check2 reporter vector, respectively. To confirm their targeting relationship, miR-216a expression plasmid and the psi-Check2 reporter vector (wild or mutant) were co-transfected into HEK293T cells. After transfection for 24 h, the relative luciferase activity was measured by using a Dual-Luciferase® Reporter DLR™Assay System Kit (Promega, USA) according to the manufacturer’s protocols.

### Immunofluorescence Staining

Immunofluorescence staining was used to detect the number of *MyHC*-positive myotubes. After transfection and induction of differentiation for 4 days, the bovine primary muscle cells were fixed with 4% paraformaldehyde in PBS for 20 min. After permeabilized with 0.5% Triton X-100 for 10 min and blocked with 5% BSA at 4°C for 30 min, the cells were incubated with primary antibody-*MyHC* diluted 1:200 with 5% bovine serum albumin (BSA) in PBS at 4°C overnight. Then the cells were incubated with the corresponding fluorescent secondary antibody [goat anti-mouse immunoglobulin G (IgG) H&L] diluted 1:400 with 5% BSA at 37°C for 2 h. Then cells were stained with 5 mg/ml 4′,6-diamidino-2-phenylindole. The cells were washed three times with PBS for 5 min before each procedure. All images were observed on a fluorescence microscope (DM5000B, Leica, Germany). The antibodies source information was listed in **Supplementary Table 2**.

### Statistical Analysis

Results were presented as mean ± SEM. The data statistical analysis was performed using one-way analysis of variance (ANOVA) or student’s t test. The significance of differences between the groups were considered significant at P <0.05 (* P < 0.05; ** P < 0.01).

## Result

### The Expression Feature of miR-216a in Bovine Tissues and Primary Muscle Cells

**Figure 1:**
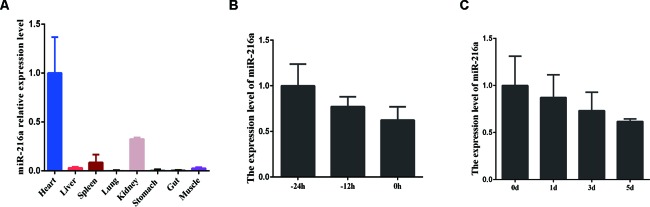
The expression feature of miR-216a in bovine tissues and primary muscle cells. **(A)** RT-qPCR analysis of relative miR-216a levels in different tissues (heart, liver, spleen, lung, kidneys, stomach, gut, and muscle) of Qinchuan cattle at embryonic stage. **(B)** RT-qPCR analysis the relative expression level of miR-216a during the growth stage (-24, -12, and 0 h) of bovine primary muscle cells. **(C)** Real-time quantitative PCR analysis the relative expression level of miR-216a during the differentiation stage (0, 1, 3, and 5 days) of bovine primary muscle cells. Data are presented as the mean ± SEM; n = 3.

To investigate the expression feature of miR-216a in various tissues (heart, liver, spleen, lung, kidney, stomach, gut, and skeletal muscle) during embryonic stage, RT-qPCR analysis data demonstrated that miR-216a was found to be mainly expressed in the heart, but relatively low in skeletal muscle (**Figure 1A**). During the growth stage (-24, -12, and 0 h) of bovine primary muscle cells, the expression level of miR-216a showed a downward trend (**Figure 1B**). During the differentiated stage (0, 1, 3, and 5 days) of bovine primary muscle cells, the expression level of miR-216a also showed a downward trend (**Figure 1C**). Together, the expression characteristics of miR-216a suggested that it might play a negative factor in the proliferation and differentiation of bovine primary muscle cells.

### miR-216a Inhibits Bovine Primary Muscle Cells Proliferation

**Figure 2:**
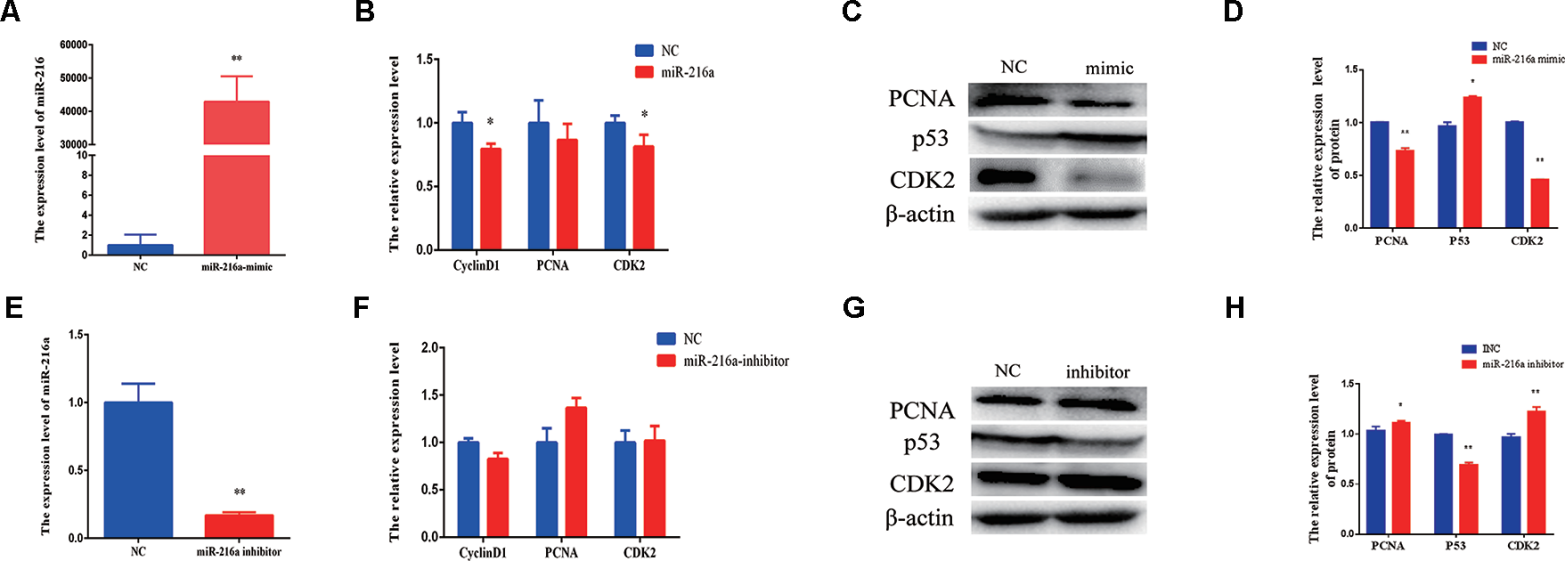
miR-216a regulates bovine primary muscle cells proliferation at the molecular level. **(A)** The detection of expression efficiency of miR-216a after transfection miR-216a mimic. **(B)** Gain of miR-216a, cell proliferation key genes *(CyclinD1, PCNA,* and *CDK2*) messenger RNA (mRNA) were detected by RT-qPCR. **(C)** Gain of miR-216a, western blot was used to detect the protein expression level of *PCNA*, *CDK2,* and *p53*. **(D)** Protein quantitative analysis of *PCNA*, *p53,* and *CDK2* for **(C)**. **(E)** The detection of expression level of miR-216a after transfection miR-216a inhibitor. **(F)** The mRNA expression of cell proliferation genes *CyclinD1*, *PCNA,* and *CDK2* were detected by real-time quantitative PCR after loss of miR-216a. **(G)** Western blot was used to detect the protein expression level of *PCNA*, *CDK2,* and *p53* after loss of miR-216a. **(H)** Protein quantitative analysis of *PCNA*, *p53,* and *CDK2* for **(G)**. Data are presented as the mean ± SEM; n = 3; *P < 0.05 and **P < 0.01.

**Figure 3:**
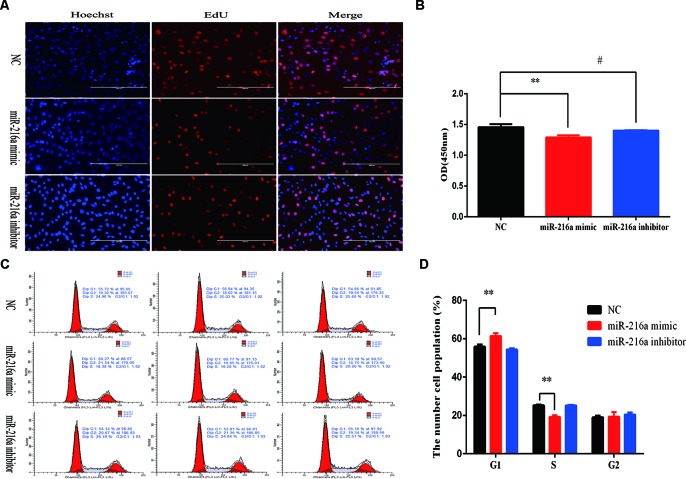
miR-216a regulates bovine primary muscle cells proliferation at the cellular level.**(A)** Bovine primary muscle cells in the S-phase were stained with 5-ethynyl-2′-deoxyuridine in red, and the cell nuclei were dyed with Hoechst in blue after transfection miR-216a mimic or inhibitor, respectively. **(B)** During bovine primary muscle cells growth stage, CCK-8 analysis was performed after gain or loss miR-216a. **(C)** Bovine primary muscle cells phases were analyzed by flow cytometry after transfected with miR-216a mimic or inhibitor, respectively. **(D)** Statistical results of flow cytometry. Data are presented as the mean ± SEM; n = 3; **P < 0.01 and ^#^ *P* > 0.05.

To verify the function of miR-216a for bovine primary muscle cells proliferation, miR-216a mimic was transfected into cells to enhance its expression level. After transfection for 24 h, RT-qPCR detection data showed that the relative expression of miR-216a was significantly higher than that of the control group (**Figure 2A**). At the molecular level, RT-qPCR was used to detect cell proliferation key genes (*CyclinD1*, *PCNA*, and *CDK2*) mRNA expression level. The analysis data demonstrated that the expression of *CyclinD1* and *CDK2* was significantly lower than that of the control group, but not *PCNA* (**Figure 2B**). Western blot analysis showed that the expression level of *PCNA* and *CDK2* protein decreased significantly and the expression level of *p53* protein increased significantly (**Figures 2C, D**). Inhibition of endogenous miR-216a expression was induced by its inhibitor (**Figure 2E**). Unfortunately, loss of miR-216a did not significantly affect the mRNA levels of the cell proliferation key genes (**Figure 2F**). But the protein levels of the cell proliferation key genes are significantly affected which consisted with overexpression (**Figures 2G, H**). At the cellular level, overexpression of miR-216a reduced the number of EdU positive cells, while no significant changes were observed after inhibition of miR-216a (**Figure 3A**). The results of CCK-8 assay showed that the overexpression of miR-216a resulted in a significant decrease in cell optical density (OD) value, while the cell OD value did not change significantly after interfering with mir-216a (**Figure 3B**). Detection the cell cycle by flow cytometry showed that overexpression of miR-216a increased the number of cells in G1 phase and decreased the number of cells in S phase, while the inhibition of miR-216a had no significant effect on cell cycle (**Figures 3C, D**). Together, the above results indicated that miR-216a inhibited the proliferation of bovine primary muscle cells.

### miR-216a Inhibits Bovine Primary Muscle Cells Differentiation

**Figure 4:**
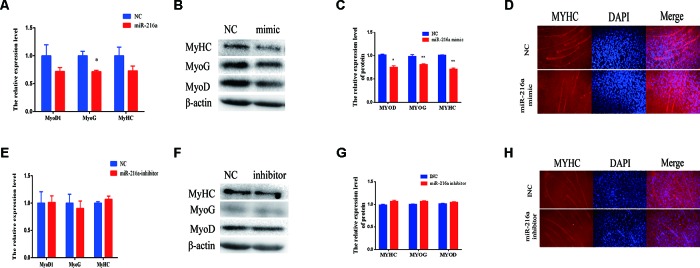
miR-216a regulates bovine primary muscle cells differentiation. **(A)** RT-qPCR was used to detect the messenger RNA (mRNA) expression of myogenic marker genes *MyoD1*, *MyoG*, and *MyHC* after transfection of miR-216a mimic. **(B)** Western blot was used to detect the protein expression level of *MyoD*, *MyoG*, and *MyHC* after transfection miR-216a mimic. **(C)** Protein quantitative analysis of *MYHC*, *MYOG*, and *MYOD* for **(B)**. **(D)** The myotube formation of bovine primary muscle cells was measured by immunofluorescence assay under the 200 times field of microscope after transfection miR-216a mimic. **(E)** After loss of miR-216a, the mRNA expression of myogenic marker genes *MyoD1*, *MyoG*, and *MyHC* were detected by RT-qPCR. **(F)** After loss of miR-216a, the protein level of *MyoD*, *MyoG*, and *MyHC* were detected by western blot. **(G)** Protein quantitative analysis of *MYHC*, *MYOG*, and *MYOD* for **(F)**. **(H)** After loss of miR-216a, the myotube formation of bovine primary muscle cells was measured by immunofluorescence assay under the 200 times field of microscope. Data are presented as the mean ± SEM; n = 3; *P < 0.05 and **P < 0.01.

When the confluence of bovine primary cells reached about 100%, horse serum at a low concentration of 2% was used to induce the differentiation into myotube. Since miR-216a regulates the proliferation of bovine primary muscle cells, it is not clear what role miR-216a plays in the differentiation process. After transfection of miR-216a mimic for 3 days, RT-qPCR was used to detect the mRNA expression level of differentiation marker gene (*MyoD1*, *MyoG*, and *MyHC*). It was found that the mRNA expression levels of *MyoD*, *MyoG*, and *MyHC* were all decreased, and the expression level of MyoG decline was significant (**Figure 4A**). Western blot results indicated that the protein expression levels of *MyoD*, *MyoG*, and *MyHC* were significantly decreased compared with the control group (**Figures 4B, C**). Immunofluorescence results showed that overexpression of miR-216a significantly inhibited the formation of myotube (**Figure 4D**). However, inhibition of miR-216a did not significantly affect the mRNA and protein levels of myoblast differentiation marker genes (**Figures 4E–G**). In addition, there was no significant difference in myotube formation after inhibition of miR-216a (**Figure 4H**). All these data demonstrated that overexpression of miR-216a inhibited bovine primary muscle cells differentiation.

### SNIP1 and *Smad7* Were the Targets of miR-216a

**Figure 5:**
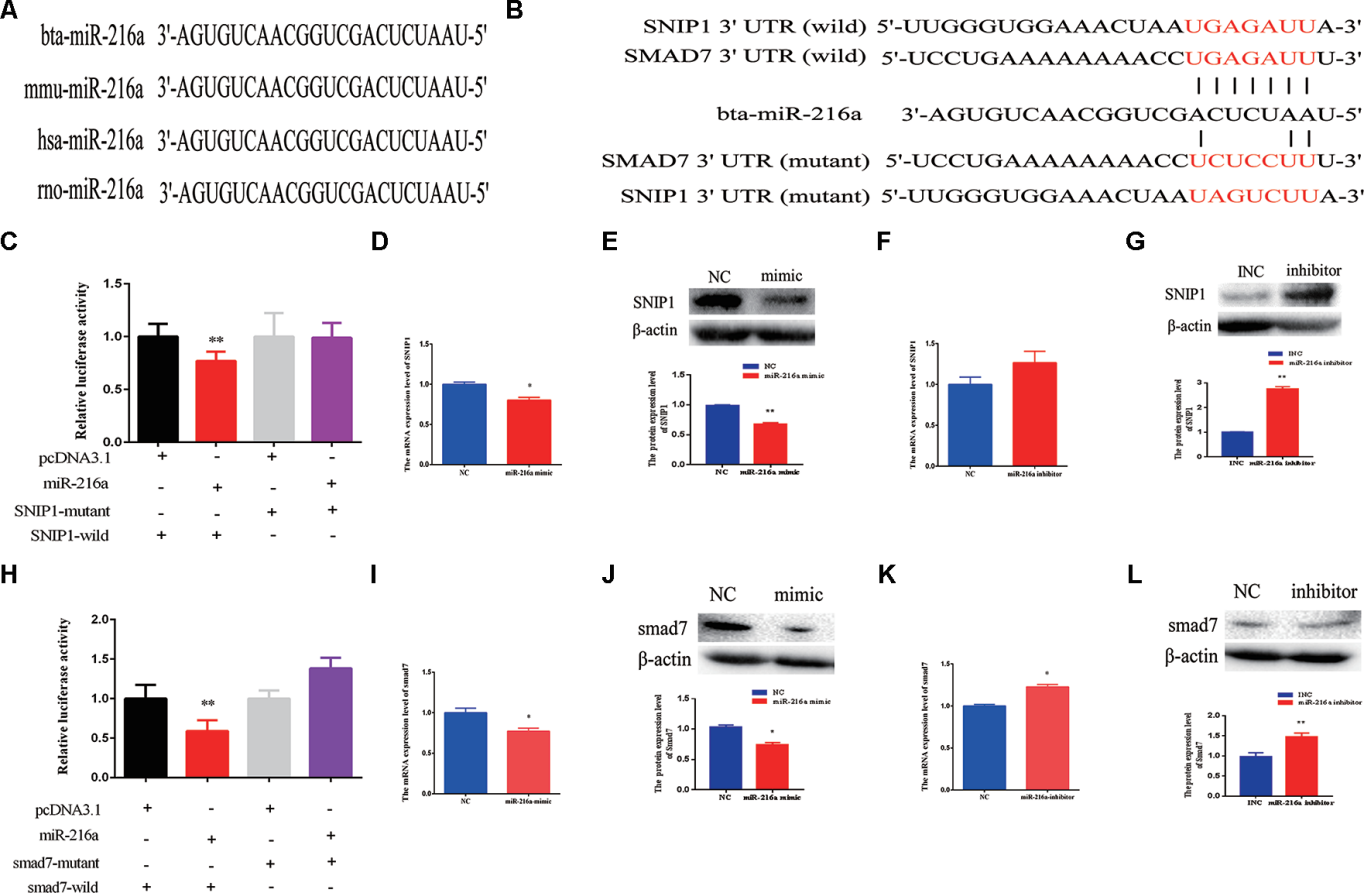
SMAD nuclear interacting protein-1 (*SNIP1*) and *smad7* were the targets of miR-216a. **(A)** Conservative analysis of the mature sequence of miR-216a among different species (bovine, mouse, human, and rat). **(B)** The binding sites of miR-216a in the 3′-UTR of *SNIP1* and *smad7* were predicted by TargetScan software. The sequences in red indicate the target position of the miR-483 seed sequence and certain mutated bases. **(C)** The relative luciferase activity was detected after transfection of miR-216a expression plasmid with *SNIP1* (wild or mutant) psi-Check2 reporter vector into HEK293T cells. **(D**, **E)** During the growth stage of bovine primary muscle cells, the messenger RNA (mRNA) and protein expression level of *SNIP1* were detected by real-time quantitative PCR (RT-qPCR) and western blot after transfection miR-216a mimic. **(F**, **G)** During the growth stage of bovine primary muscle cells, the mRNA and protein expression level of *SNIP1* were detected by RT-qPCR and western blot after transfection miR-216a inhibitor. **(H)** The relative luciferase activity was detected after transfection of miR-216a expression plasmid with *smad7* (wild or mutant) psi-Check2 reporter vector into HEK293T cells. **(I**, **J)** During the differentiation stage of bovine primary muscle cells, the mRNA and protein expression level of *samd7* were detected by RT-qPCR and western blot after transfection miR-216a mimic. **(K**, **L)** During the differentiation stage of bovine primary muscle cells, the mRNA and protein expression level of *samd7* were detected by RT-qPCR and western blot after transfection miR-216a inhibitor. Data are presented as the mean ± SEM; n = 3; *P < 0.05 and **P < 0.01.

Further, we explored molecular mechanisms underlying the function of miR-216a in impeding bovine primary muscle cells proliferation and differentiation by investigating its target genes. The mature sequence of miR-216a was highly conservative among different species (bovine, mouse, human, and rat) (**Figure 5A**), which aroused our research interest. To validate the function of miR-216a for bovine muscle cells proliferation and differentiation, predicting and verifying its target genes is essential. Through the online prediction software TargetScan (http://www.Targetscan.org) the seed sequence of miR-216a can target the (3’UTR) of the gene *SNIP1* and *smad7* mRNA (**Figure 5B**). After transfection of miR-216a expression plasmid with *SNIP1* (wild or mutant) and *smad7* (wild or mutant) psi-Check2 reporter vector into HEK293T cells, respectively, the analysis results showed that miR-216a could reduce the luciferase activity of their wild-type reporter vector but has no effect on the mutant reporter vector (**Figures 5C, H**). During the growth stage of bovine skeletal muscle cells, gain of miR-216a could reduce the mRNA and protein levels of *SNIP1* significantly (**Figures 5D, E**). At the same time, miR-216a inhibitor was transfected into the cells, which had significantly enhanced the mRNA and protein levels of *SNIP1* (**Figures 5F, G**). During the differentiated stage of bovine skeletal muscle cells, gain of miR-216a reduced *smad7* mRNA and protein expression level (**Figures 5I, J**), whereas loss of miR-216a enhanced *smad7* mRNA and protein expression level (**Figures 5K, L**). According to the above results, during the proliferation and differentiation stages of bovine primary muscle cells, miR-216a targeted *SNIP1* and *smad7*, respectively.

## Discussion

In this study, we found that miR-216a possessed an inhibitory role in the process of proliferation and differentiation of bovine primary muscle cells by targeting *SNIP1* and *smad7*, respectively. Herein, miRNA-216a as a non-muscle-specific miRNA presented a downward trend in the proliferative and differentiation stages of bovine primary muscle cells, which suggested a potential role in the development of bovine skeletal muscle. To evaluate this hypothesis, mimic and inhibitor of miR-216a were used to enhance or block its expression, respectively. The current research found that overexpression of miR-216a can inhibit bovine primary muscle cells proliferation and differentiation. Unfortunately, inhibition of miR-216a expression did not affect the proliferation and differentiation of bovine primary muscle cells. We speculated that this might be caused by the low expression of miR-216a in bovine skeletal muscle or other potential compensation mechanisms. And it has been reported that an individual miRNA might interact with hundreds of mRNA targets while individual mRNAs can be targeted by many different miRNAs (Sassen et al., 2008). It was observed that if one specific miRNA fails, the pathway might be regulated by another miRNA within a complex cross path of miRNA network (Rottiers et al., 2011; Ebert and Sharp, 2012; Lima et al., 2017).

In the current study, we found miR-216a played a significant role in inhibiting skeletal muscle growth and development. According to our prediction and analysis, the mature sequence of miR-216a was found to be highly conserved among different species (bovine, human, mouse, and rat) (**Figure 5A**). Consistent with the research results, in previous studies, miR-216a also mainly played a suppressive role in disease and tumorigenesis. For instance, miR-216a inhibited tumor cells growth by down-regulating the expression level of Janus kinase 2 (*JAK2*) in pancreatic cancer (Siliang et al., 2014; Hou et al., 2016). In colorectal cancer, miR-216a can impede tumor cells invasion *in vitro* and metastasis *in vivo* by down-regulation of *KIAA1199* (Zhang et al., 2017). In gastric cancer, miR-216a restrained tumor cells migration and invasion possibly by targeting *JAK2/STAT3*-mediated epithelial-mesenchymal transition (EMT) (Tao et al., 2017). In osteosarcoma patients, the expression level of miR-216a was downregulated. Gain of miR-216a can inhibit tumor cells proliferation, migration and invasion *in vitro* and *in vivo via* suppressing the expression of *CDK14* (Ji et al., 2017). In renal cell carcinoma, miR-216a exerts tumor-suppressing functions by targeting *TLR4* (Wang et al., 2018c). Based on the above studies, it’s worth noting that miR-216a has a broad application prospect in cancer therapy. Besides these, in muscle, a certain preliminary study of miR-216a has been reported. For example, miR-216a inhibits proliferation and promotes apoptosis of human airway smooth muscle cells by targeting *JAK2* (Yan et al., 2019). And it can also exacerbate *TGF-β*-induced myofibroblast trans-differentiation *via PTEN/AKT* signaling (Qu et al., 2019). These results suggest that miR-216a has a potentially important role in muscle development. So, it is important to explore its role in skeletal muscle growth and development.

The current study further confirmed that miR-216a regulated the proliferation and differentiation of bovine primary muscle cells by targeting *SNIP1* and *smad7.* Bioinformatics prediction, dual-luciferase reporter assay, and related verification suggested that *SNIP1* and *smad7* were the direct targets of miR-216a. In previous studies, *SMAD nuclear interacting protein-1* (*SNIP1*) had been demonstrated function as an oncogene (Fujii et al., 2006), which can partially regulate *CyclinD1* to accelerate cell cycle progression through G1 (Roche et al., 2004; Etsu et al., 2010). In a variety of cellular contexts, knockdown of *SNIP1* could inhibit the mRNA and protein levels of *CyclinD1* (Roche et al., 2004; Bracken et al., 2008). In the current study, overexpression of mir-216a in the growth stage of bovine primary muscle cells significantly inhibited the mRNA and protein levels of *SNIP1*, and the mRNA expression level of *CyclinD1* also decreased at the same stage. *Smad7*, as a member of the inhibitory Smads (I-Smads) family, can specifically inhibit *TGF-β* pathway as described above and can also inhibit myostatin signaling by competitive combination with the R-Smads (Xiangyang et al., 2004). It has been reported that *TGF-β* signal pathway participated in regulating satellite cells (myoblasts) proliferation and differentiation by a complex regulatory network (Greene and Allen, 1991; Hathaway et al., 1991; Liu et al., 2001; Allen and Boxhorn, 2010). It has been reported that *Smad7* can promotes and enhances skeletal muscle differentiation not only by blocking smad2/3 transcriptional activity but also by physically interacting with *MyoD in vitro* experiments (Kollias et al., 2006). *Smad7-/-* mice have the feature of reducing muscle cells proliferation and differentiation by enhancing *smad2/3* signaling, which impedes muscle growth and regeneration (Cohen et al., 2015).

In summary, the current experimental results indicated that miR-216a suppressed bovine primary muscle cells proliferation and differentiation. Bioinformatics analysis, dual luciferase report analysis and experimental verification showed that *SNIP1* and *smad7* were the targets of miR-216a regulating the proliferation and differentiation of bovine muscle primary cells, respectively. Therefore, the function and mechanism of miR-216a in bovine primary muscle cells growth might provide theoretical basis for elucidating bovine skeletal muscle development in future experiments.

## Data Availability Statement

All datasets generated for this study are included in the article/**Supplementary Material**.

## Ethics Statement

Animal care and study protocols were approved by the Animal Care Commission of the College of Veterinary Medicine, Northwest A&F University (Permit Number: NWAFAC1019).

## Author Contributions

ZY and CS contributed equally to this work. ZY conducted the analysis and wrote the manuscript. CS designed the study and finalized the manuscript. RJ carried out the part of experiments. YH, XL, CL, and HC provided experimental guidance.

## Funding

This work was supported by the National Natural Science Foundation of China No.31772574), the Program of National Beef Cattle and Yak Industrial Technology System (CARS-37).

## Conflict of Interest

The authors declare that the research was conducted in the absence of any commercial or financial relationships that could be construed as a potential conflict of interest.
